# Generation and characterization of new monoclonal antibodies targeting the PHF1 and AT8 epitopes on human tau

**DOI:** 10.1186/s40478-017-0458-0

**Published:** 2017-07-31

**Authors:** Kevin H. Strang, Marshall S. Goodwin, Cara Riffe, Brenda D. Moore, Paramita Chakrabarty, Yona Levites, Todd E. Golde, Benoit I. Giasson

**Affiliations:** 1 Department of Neuroscience, College of Medicine University of Florida, Gainesville, FL 32610 USA; 2 Center for Translational Research in Neurodegenerative Disease, College of Medicine University of Florida, Gainesville, FL 32610 USA; 3 McKnight Brain Institute, College of Medicine University of Florida, Gainesville, FL 32610 USA; 4 BMS J483/CTRND, 1275 Center Drive, Gainesville, FL 32610 USA

**Keywords:** Alzheimer’s disease, Antibodies, Phosphorylation, Tau, Transgenic mice

## Abstract

Tauopathies are a group of neurodegenerative disorders, including Alzheimer’s disease, defined by the presence of brain pathological inclusions comprised of abnormally aggregated and highly phosphorylated tau protein. The abundance of brain tau aggregates correlates with disease severity and select phospho-tau epitopes increase at early stages of disease. We generated and characterized a series of novel monoclonal antibodies directed to tau phosphorylated at several of these phospho-epitopes, including Ser396/Ser404, Ser404 and Thr205. We also generated phosphorylation independent antibodies against amino acid residues 193–211. We show that most of these antibodies are highly specific for tau and strongly recognize pathological inclusions in human brains and in a transgenic mouse model of tauopathy. They also reveal epitope-specific differences in the biochemical properties of Alzheimer’s disease sarkosyl-insoluble tau. These new reagents will be useful for investigating the progression of tau pathology and further as tools to target the cellular transmission of tau pathology.

## Introduction

Tau refers to the microtubule-associated protein [10, 47] expressed from the *MAPT* gene located on chromosome 17q21–22 [1, 39]. In the adult human brain, 6 major tau isoforms ranging between 352 and 441 amino acids in length are produced as a result of alternative RNA splicing [20, 21]. Although tau is natively highly soluble [11, 43], it can polymerize to form amyloid fibrils [16] that aberrantly coalesce to form intracellular inclusions that define a large spectrum of neurodegenerative diseases termed tauopathies [15, 29, 31, 46].

Alzheimer’s disease (AD) is the most common form of tauopathy where tau aggregates to form somatodendritic neurofibrillary tangles (NFTs), parenchymal neuropil threads and dystrophic neurites that are intertwined with extracellular deposits of amyloid-β peptides [15, 29, 31, 46]. The abundance of tau inclusions in the brain correlates well with disease severity [2]. However, the presence of tau pathological inclusions are also a defining feature of many other types of dementias, such as corticobasal degeneration, progressive supranuclear palsy, tangle-only dementia, Pick’s disease, and frontotemporal dementia and parkinsonism linked to chromosome 17 with tau pathology (FTDP-17 t) [15, 17, 28, 31]. Indeed, the fact that FTDP-17 t is caused by more than 50 different mutations in the *MAPT* gene, resulting in either tau protein amino acid changes or altered ratio of tau splicing isoforms, is irrefutable evidence for a pathogenic role of tau in neurodegeneration [17, 28].

Hyperphosphorylation of tau is a hallmark of tau pathological inclusions in human brains [24–26, 38] and pathological findings indicate that phosphorylation of tau at specific residues, such as the AT8 and PHF1 epitopes, occurs early in tau inclusion formation [8, 9, 37]. Given the interest for these epitopes as pathological markers and for immunotherapy [3, 4, 6, 7, 13, 22, 30, 45], we have generated and characterized a series of new monoclonal antibodies targeting these regions of tau with unique phosphorylation specificities.

## Materials and methods

### Mice

Tau null (tau KO) mice [14] and tau transgenic (Tg) mice line PS19 expressing human 1 N/4R tau with the P301S mutation driven by the mouse prion promoter [49] were obtained from Jackson Laboratory (Bar Habor, ME). Tau Tg mice line JNPL3 expressing human 0 N/4R tau with the P301L mutation were previously described [33].

### Antibodies

AT8 (Thermo-fisher) is a mouse monoclonal antibody specific towards phosphorylation sites S202 and T205 in tau [19] that can also be influenced by phosphorylation at S199 or S208 [34, 42]. PHF1 (generously provided by Dr. Peter Davies, Albert Einstein University, NY, NY) is a mouse monoclonal antibody specific towards phosphorylation sites S396 and S404 in tau [40]. Rabbit polyclonal antibody (H-150) raised against the first 150 amino acids of human tau was obtained from Santa Cruz Biotechnologies (Dallax, TX), and rabbit polyclonal antibodies 3026 and 3029 raised against recombinant full-length 0 N/3R tau was generated as a service by GenScript USA Inc. (Piscataway, NJ).

### Generation of new mouse tau monoclonal antibodies

All procedures were performed according to the NIH Guide for the Care and Use of Experimental Animals and were approved by the University of Florida Institutional Animal Care and Use Committee. Phosphopeptides EIVYKpSPVVSGDTpSPRHLSC (p391–409) and DRSGYSpSPGpSPGpTPGSRSRC (p193–211) corresponding, respectively, to residues 391–409 and 193–211 in the 2 N/4R human tau isoform with a C residue added at the carboxy-termini for chemical conjugation were synthesized and purified by GenScript USA Inc. (Piscataway, NJ). Lyophilized peptides were reconstituted in phosphate buffered saline (PBS) and conjugated to Imject maleimide-activated mariculture keyhole limpet hemocyanin (mcKLH; Thermo Scientific, Waltham, MA). 2–3 month old female BALB/c mice (Jackson Laboratory, Bar Harbor, ME) were used for immunization. Injection solutions emulsified by vortexing for 15 min were prepared by combining 100 μg KLH-conjugated peptide in 200 μl PBS with 100 μl of Freund’s complete adjuvant (Sigma Aldrich, St. Louis, MO) for the initial subcutaneous injection. Three weeks following the initial injection, mice were boosted with an intraperitoneal (IP) injection of 50 μg KLH-conjugated peptide in 200 μl PBS emulsified with 100 μl of Freunds incomplete adjuvant (Sigma Aldrich, St. Louis, MO). Three weeks later, mice were boosted with an IP injection of 50 μg KLH-conjugated peptide in PBS. Three days later, mice were euthanized and spleens were harvested using aseptic technique.

Mouse myeloma (Sp2/O-Ag14; ATCC, Manassas, VA) cells were maintained in high glucose (4.5 g/L) Dulbecco’s Modified Eagle Medium (DMEM) with 10% NCTC 135 Media (Sigma Aldrich, St. Louis, MO), 20% hybridoma grade fetal bovine serum (FBS; Hyclone, Logan, UT), 100 U/ml penicillin, 100 U/ml streptomycin, 2 mM L-glutamine, 0.45 mM pyruvate, 1 mM oxaloacetate, and 0.2 U/ml insulin at 37 °C and 8% CO_2_. Spleens were gently homogenized in 5% FBS/Hank’s balanced salt solution (HBSS; Lonza, Walkersville, MD) and centrifuged to pellet cells. The cell pellet was resuspended in red blood cell lysis buffer for one minute (Sigma Aldrich, St. Louis, MO) and diluted with HBSS. The cells were then washed twice by centrifuging at 100 x g for 10 min and resuspended in HBSS. Sp2/O-Ag14 cells were also washed twice with HBSS. Five million Sp2/O-Ag14 cells were added to 50 million spleen cells, and after centrifuging at 100 x g for 10 min onto a culture dish, fusion was induced with 50% polyethylene glycol 1450 (Sigma Aldrich, St. Louis, MO). After washing with HBSS, cells were incubated in Sp2/O-Ag14 media at 37 °C with 8% CO_2_ overnight. The next day, the cells were gently detached from the plate and distributed into 96 well plates with Sp2/O-Ag14 media/0.5% hybridoma enhancing supplement (Sigma Aldrich, St. Louis, MO)/HAT selection supplement (Sigma Aldrich, St. Louis, MO).

### Hybridoma screening

All hybridoma clones were screened for reactivity to the respective unconjugated peptide that was used for immunization by enzyme-linked immunosorbent assay (ELISA). MaxiSorp plates (Thermo Scientific, Waltham, MA) were coated with 1 μg/ml peptide in PBS and blocked with 5% FBS/PBS. Media from the hybridomas was applied to plates, which were then incubated at room temperature. Next, the plates were washed with PBS and incubated with goat anti-mouse secondary antibody conjugated to horseradish peroxidase (HRP; Jackson Immuno Research Labs, West Grove, PA) at room temperature. Then, plates were washed and TMB substrates (Pierce, Rockford, IL) were applied until color changes were observed. Reactions were then quenched with 1 M HCl and absorbance was measured at 450 nm. Clones that were positive by ELISA were transferred to larger culture plates as needed. The positive clones were next screened by immunohistochemistry of a human AD autopsy case with abundant tau pathology.

Antibody clones were isotyped with the mouse monoclonal antibody isotyping kit purchased from Sigma-Aldrich (St. Louis, MO).

### Immunohistochemistry analyses

Paraffin embedded, formalin fixed human brain tissue was obtained through the University of Florida Neuromedicine Human Brain Tissue Bank. Sequential tissue sections were deparaffinized with xylenes and sequentially rehydrated with graded ethanol solutions (100–70%). Antigen retrieval was performed by incubating sections in 0.05% Tween-20 in a steam bath for 60 min. Endogenous peroxidase activity was quenched with 1.5% hydrogen peroxide/0.005% Triton-X-100/PBS for 20 min. Following washes, sections were blocked with 2% FBS/0.1 M Tris, pH 7.6 and incubated with primary antibody overnight at 4 °C. Following washing with 0.1 M Tris, pH 7.6, sections were incubated with biotinylated horse anti-mouse IgG secondary antibody (Vector Laboratories, Burlingame, CA) diluted in 2% FBS/0.1 M Tris, pH 7.6 for 1 h. Next, sections were washed with 0.1 M Tris, pH 7.6, then incubated with streptavidin-conjugated HRP (VECTASTAIN ABC kit; Vector Laboratories, Burlingame, CA) diluted in 2% FBS/0.1 M Tris, pH 7.6 for 1 h. Sections were washed with 0.1 M Tris, pH 7.6, and then developed with 3, 3′- diaminobenzidine (DAB kit; KPL, Gaithersburg, MD). Reactions were stopped by immersing the slides in 0.1 M Tris, pH 7.6, and sections were counterstained with Mayer’s hematoxylin (Sigma Aldrich, St. Louis, MO). Next, sections were dehydrated with an ascending series of ethanol solutions (70%–100%) followed by xylenes and coverslipped using cytoseal (Thermo Scientific, Waltham, MA).

### Recombinant tau protein production and purification

Human full-length tau cDNA (0N/3R or 2N/4R isoform) cloned into the bacterial expression vector pRK172 were kindly provided by Dr. Michel Goedert. pRK172 plasmid expressing human 0 N/3R tau with the S199A, T202A or T205A mutations (numbered according to the 2 N/4R tau isoform) or human 2 N/4R tau with the S396A or S404A mutations (numbered according to the 2 N/4R tau isoform) was created with oligonucleotides corresponding to the amino acid substitutions by QuickChange site-directed mutagenesis (Stratagene, La Jolla, CA). These recombinant tau proteins were expressed in *E. coli* BL21 and purified, as previously described [18, 27].

### Kinase reactions

Tau proteins (0.2 mg/ml) were phosphorylated with either glycogen synthase kinase 3β (GSK3β; 2.5 U/ul; New England Biolabs, Ipswich, MA) or p42 mitogen-activated protein kinase (MAPK; 0.5 U/ul; New England Biolabs, Ipswich, MA) in 50 mM Tris, pH 7.5, 10 mM MgCl_2_, 0.1 mM EDTA, 2 mM DTT, 0.1% Brij 35, 200 uM ATP for 2 h at 30 °C followed by heat inactivation at 95 °C for 5 min. Samples were diluted in SDS-sample buffer and 100 ng of each tau protein was loaded on a separate lane on SDS-polyacrylamide gels for immunoblot analysis.

### Preparation of total mouse brain protein lysates

Tau KO, PS19 Tg or non-transgenic (nTg) mice were humanely euthanized and the brains were harvested. Brain tissue was lysed with 2% SDS/50 mM Tris, pH 7.5 using a probe sonicator until homogenous and incubated for 10 min at 100 °C. Protein concentrations were determined by BCA assay (Thermo Scientific) using bovine serum albumin (BSA) as the standard. SDS sample buffer was added, and equal amounts of protein (40 μg) were resolved by SDS-PAGE and analyzed by immunoblot.

### Preparation of sarkosyl-insoluble human temporal cortex samples

Frozen human brain tissue was obtained through the University of Florida Neuromedicine Human Brain Tissue Bank. Pulverized temporal cortex tissue from human AD cases (*n* = 3) or control (*n* = 2) was homogenized in 3 ml of high-salt (HS) buffer (50 mM Tris–HCl, pH 7.5, 0.75 M NaCl, 2 mM EDTA, 50 mM NaF with a cocktail of protease inhibitors) per gram of tissue and sedimented at 100,000 x g for 30 min at 4 °C. Supernatants were collected (HS fraction) and pellets were re-suspended in 2 ml of HS buffer containing 1% Triton X-100 per gram of tissue. Samples were sedimented at 100,000 x g for 30 min at 4 °C and the supernatants were collected (HS/Triton-soluble fraction). Pellets were again re-extracted in HS buffer/1% Triton X-100. Samples were sedimented at 100,000 x g for 30 min at 4 °C and supernatants were discarded. Pellets were re-suspended in 1 ml of HS buffer containing 1% sarkosyl per gram of tissue, incubated at 37 °C for 30 min, and sedimented at 100,000 x g for 30 min at 4 °C. Supernatants were collected (sarkosyl-soluble fraction). The detergent-insoluble pellets were extracted in 0.5 ml of 4 M urea, 2% SDS, 25 mM Tris–HCl pH 7.6 per gram of tissue, sonicated, and sedimented at 100,000 x g for 30 min at 25 °C. Protein concentrations were determined by BCA assay (Thermo Scientific) using BSA as the standard. SDS sample buffer was added, and equal amounts of protein (10 μg) were resolved by SDS-PAGE and analyzed by immunoblot.

### Immunoblotting analyses

Protein samples were resolved by electrophoresis on 10% polyacrylamide gels, then electrophoretically transferred to nitrocellulose membranes. Membranes were blocked with 5% milk/Tris-buffered saline (TBS) and then incubated overnight at 4 °C with primary antibodies diluted in 5% BSA/TBS. Following washing, blots were incubated with HRP conjugated goat anti-mouse IgG/IgM (heavy and light chains, but pre-absorbed to human, bovine and horse serum proteins) or goat anti-rabbit secondary antibodies (Jackson Immuno Research Labs, West Grove, PA) diluted in 5% milk/TBS for 1 h. Following washing, protein bands were visualized using Western Lightning-Plus ECL reagents (PerkinElmer, Waltham, MA), and images were captured using the GeneGnome XRQ system and GeneTools software (Syngene, Frederick, MD).

## Results

### Generation and characterization of anti-phospho antibodies targeted to S396/S404 in tau

**Fig. 1:**
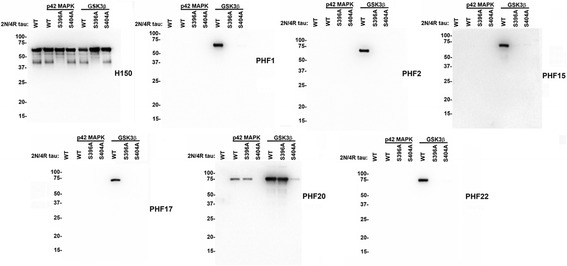
Specificity of novel tau antibodies raised against tau peptide p391–409 as determined by immunoblotting with recombinant tau proteins phosphorylated in vitro with p42 MAPK or GSK3β. WT, S396A and S404A 2 N/4R tau were incubated with p42 MAPK or GSK3β or without any kinase as described in “Material and Methods.” The proteins were resolved onto 10% polyacrylamide gels and analyzed by immunoblotting with novel tau antibodies (as indicated for each *blot*) PHF2, PHF15, PHF17, PHF20 or PHF22. *Similar blots* were performed with previously characterized antibody PHF1 and total tau antibody H150. The mobilities of molecular mass markers are shown on the *left*

To generate antibodies similar to the PHF1 epitope [40] we immunized mice with the synthetic phosphopeptide EIVYKpSPVVSGDTpSPRHLSC corresponding to residues 391–409 in 2 N/4R human tau and containing phosphorylated S396 and S404. Several hybridomas, termed the PHF series, were identified by ELISA screening as well as initial screening for reactivity of tau pathology by immunohistochemistry of a human AD autopsy case with abundant tau pathology. Five hybridomas (PHF2, PHF15, PHF17, PHF20 and PHF22) were identified using these criteria. To determine the specificity of these monoclonal antibodies, we in vitro phosphorylated full-length WT, S396A and S404A 2 N/4R human tau with known tau kinases (p42 MAPK or GSK3β) and used these tau proteins for immunoblotting analysis (Fig. 1). We used the previously characterized antibody PHF1, that recognizes tau phosphorylated at both S396 and S404, as a control [40]. WT but not S396A or S404A tau phosphorylated with GSK3β reacted with PHF1 as expected. PHF20 reacted with WT or S396A but not S404A tau phosphorylated with either GSK3β or p42 MAPK, demonstrating that this antibody preferentially recognizes tau phosphorylated at S404. Since PHF1 did not react with tau phosphorylated with p42 MAPK these data also show that p42 MAPK in vitro phosphorylated tau only at S404 and not S396. Antibodies PHF2, PHF15, PHF17 and PHF22 all revealed the same relativities as PHF1 in these assays, demonstrating that they recognize tau only when phosphorylated at both S396 and S404.

**Fig. 2:**
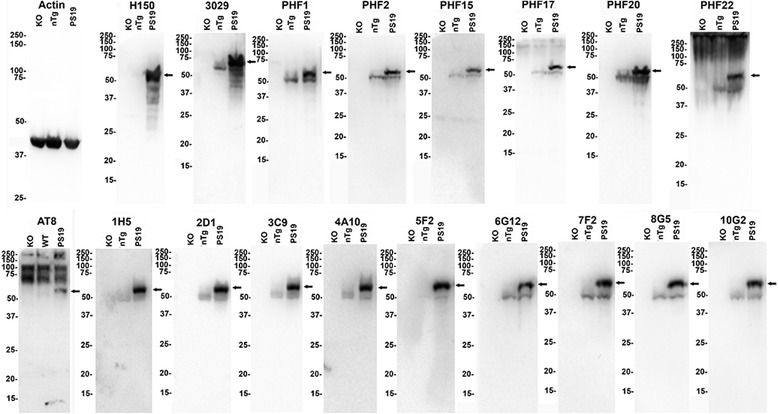
Characterization of the specificity of novel tau antibodies by immunoblotting analyses using total brain lysates from nTg, tau KO and PS19 tau Tg mice. Brains from tau KO, nTg, and PS19 tau Tg mice were harvested and lysed in 2% SDS/50 mM Tris, pH 7.5 as described in “Material and Methods.” Equal amounts of proteins (40 μg) from each sample was resolved onto 10% polyacrylamide gels and analyzed by immunoblotting with each antibody indicated above. *Arrows* depict human tau expressed in PS19 mice. The mobilities of molecular mass markers are shown on the *left*

The specificity of the new PHF series of antibodies was then assessed using total mouse brain lysates from tau KO, nTg and PS19 tau Tg mice (Fig. 2). Antibodies PHF2, PHF15, PHF17 and PHF 20 specifically reacted with mouse tau in nTg mice and with both mouse and human tau in lysates derived from PS19 tau Tg mice. PHF2, PHF15, and PHF 20 displayed no cross-reactivity in brain extracts from tau KO mice, but PHF17 weakly cross-reacted with a ~150 kDa non-tau protein band. PHF22 recognized mouse and human tau, but it also cross-reacted with some non-specific higher molecular mass proteins still present in lysate from tau KO mice.

### Generation and characterization of anti-tau antibodies targeting the phospho-S199/S202/T205 tau epitope

**Fig. 3:**
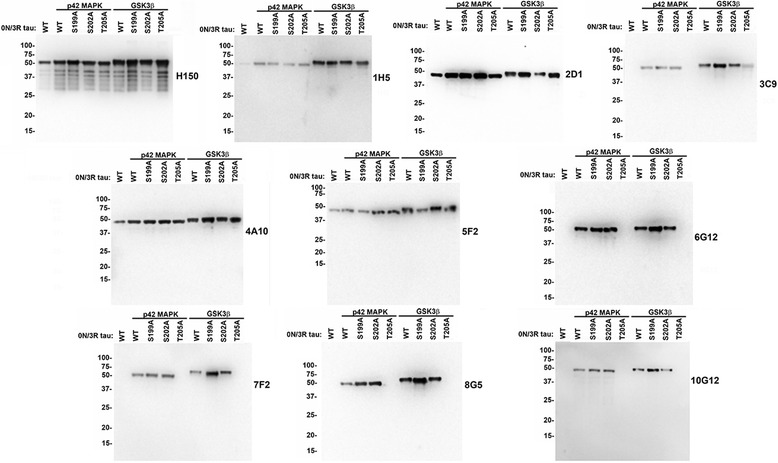
Specificity of novel tau antibodies raised against tau peptide p193–211 as determined by immunoblotting with recombinant tau proteins phosphorylated in vitro with p42 MAPK or GSK3β. WT, S199A, S202A and T205A 0 N/3R tau (mutants numbered according to 2 N/4R human tau) were incubated with p42 MAPK or GSK3β or without any kinase as described in “Material and Methods.” The proteins were resolved onto 10% polyacrylamide gels and analyzed by immunoblotting with novel tau antibodies (as indicated for each *blot*) 1H5, 2D1, 3C9, 4A10, 5F2, 6G12, 7F2, 8G5, and 10G12 and total tau antibody H150. The mobilities of molecular mass markers are shown on the *left*

Next, in an attempt to generate antibodies similar to the AT8 epitope, we immunized mice with the synthetic phosphopeptide DRSGYSpSPGpSPGpTPGSRSRC corresponding to residues 193–211 in 2 N/4R human tau with phosphorylated S199, S202 and T205. Several hybridomas were identified by ELISA screening with the corresponding unconjugated peptide followed by immunohistochemistry for tau pathology in a human AD autopsy case. Nine of these hybridomas (1H5, 2D1, 3C9, 4A10, 5F2, 6G12, 7F2, 8G5 and 10G12) were further characterized. To determine the specificity of these monoclonal antibodies, we phosphorylated WT, S199A, S202A and T205A 0 N/3R human tau (mutations numbered according to the sequence of 2 N/4R human tau) with p42 MAPK or GSK3β and performed immunoblotting analysis (Fig. 3). Antibodies 6G12, 7F2, 8G5 and 10G12 reacted with tau phosphorylated with either p42 MAPK or GSK3β, but not when T205 was mutated to an A, indicating that they all recognize tau phosphorylated at T205. Antibodies 2D1, 4A10 and 5F2 reacted with phosphorylated and non-phosphorylated tau showing that their recognition epitopes are phosphorylation independent. Antibody 1H5 reacted with phosphorylated tau but could also recognize non-phosphorylated tau to a lesser degree, demonstrating that it prefers phosphorylated tau. Antibody 3C9 only recognized phosphorylated tau and prefers tau phosphorylated at T205, as shown by the reduced reactivity with phosphorylated T205A tau. However, 3C9 is a more promiscuous phospho-antibody that can also recognize tau phosphorylated at either S199 or S202 since the T205A mutation does not completely block its reactivity with phosphorylated tau.

The specificity of the antibodies generated to the AT8 epitope were then assessed using mouse brain lysates from tau KO, nTg and PS19 tau transgenic mice (Fig. 2). All of these antibodies could detect endogenous mouse tau in nTg mice and both mouse and human tau in PS19 Tg mice, and they were quite specific for tau, as demonstrated by their lack of reactivity to lysates from tau KO mice. They are much more specific than AT8, which reacted with many additional higher molecular mass protein bands present in lysates from tau KO mice (Fig. 2). The same cross-reactivity of the AT8 antibody with non-tau proteins was observed in three independent lots of the AT8 antibody.

**Fig. 4:**
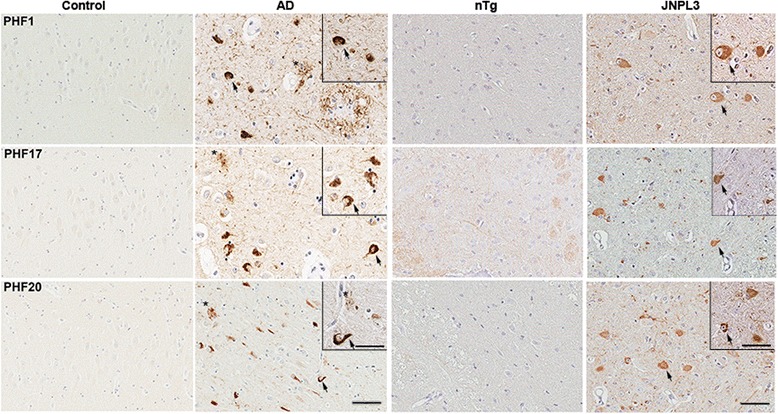
Immunocytochemistry of representative tau pathology in human AD brain and JNPL3 Tg mice with new antibodies PHF17 and PHF20. Immuno-reactivity of previously characterized phospho-tau antibodies PHF1 and new tau antibodies PHF17 or PHF20 in the hippocampus of a control individual or a subject with AD, and in the spinal cord of 12 month old nTg and JNPL3 Tg mice. *Arrows* indicating NFTs in human brain or NFT-like inclusion pathology in JNPL3 mice. *Asterisk* depicting dystrophic neurites within senile plaques. Bar = 100 μm, and 200 μm for *insets*

**Fig. 5:**
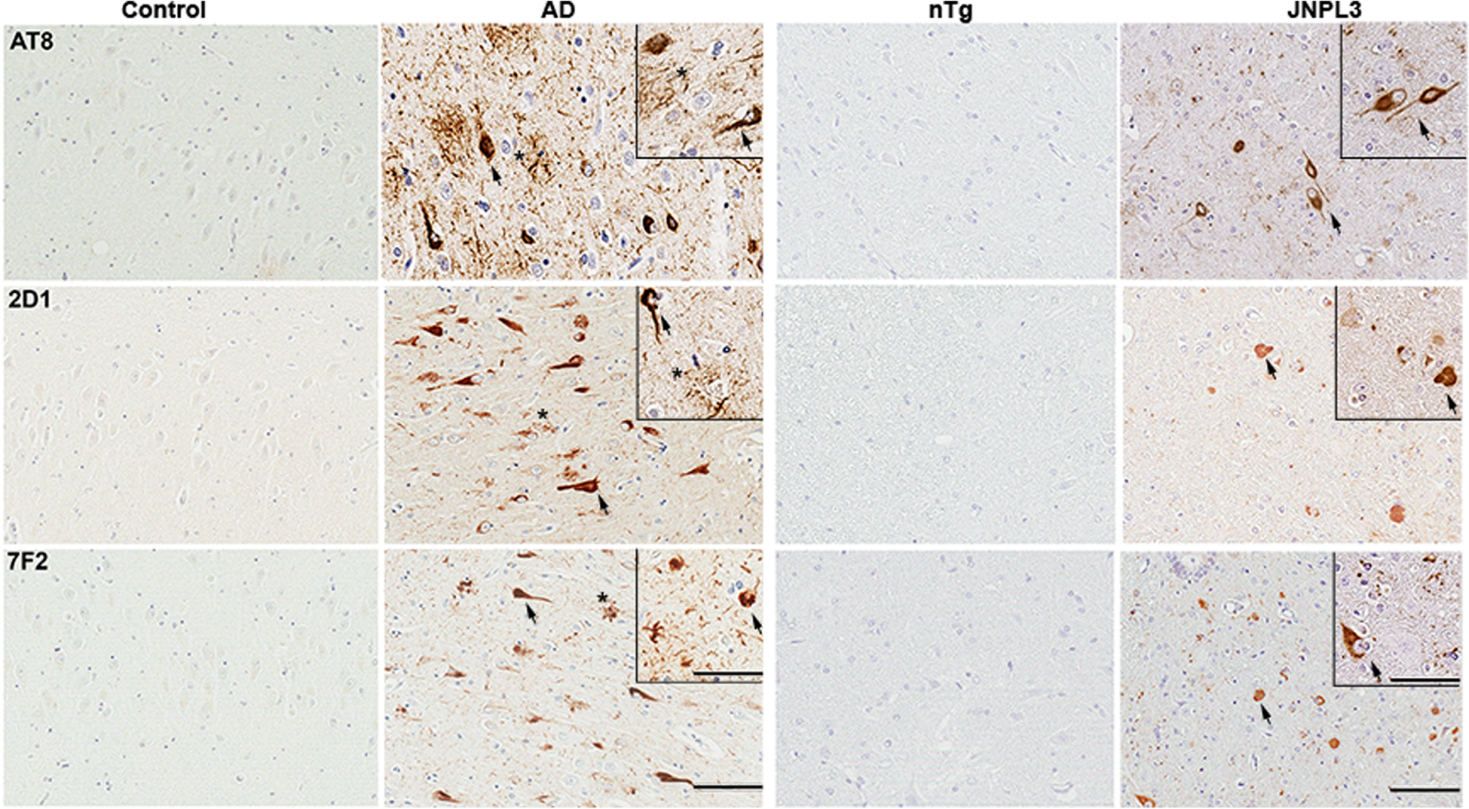
Immunocytochemistry of representative tau pathology in human AD brain and JNPL3 Tg mice with new antibodies 2D1 and 7F2. Immuno-reactivity of previously characterized phospho-tau antibodies AT8 and new tau antibodies 2D1 or 7F2 in the hippocampus of a control individual or a subject with AD, and in the spinal cord of 12 month old nTg and JNPL3 Tg mice. *Arrows* indicating NFTs in human brain or NFT-like inclusion pathology in JNPL3 mice. *Asterisk* depicting dystrophic neurites within senile plaques. Bar = 100 μm, and 200 μm for *insets*

All of the new tau antibodies were compared for immunoreactivity of tau pathology using brain tissue from AD and control patients. All of these antibodies reacted with NFTs, but antibodies PHF17, PHF20, PHF22, 2D1 and 7F2 yielded the strongest staining with little background (Figs. 4 and 5; data not shown); however, as shown in the immunoblotting screen, PHF22 is not completely tau specific (Fig. 2). Antibodies 2D1 and 7F2 also provided strong detection of neuropil threads in addition to senile plaque dystrophic neurites. Antibodies PHF17, PHF20, 2D1 and 7F2 were also tested for immunoreactivity of tau pathology in the JNPL3 Tg mouse model, where they showed strong immunoreactivity (Figs. 4 and 5).

**Fig. 6:**
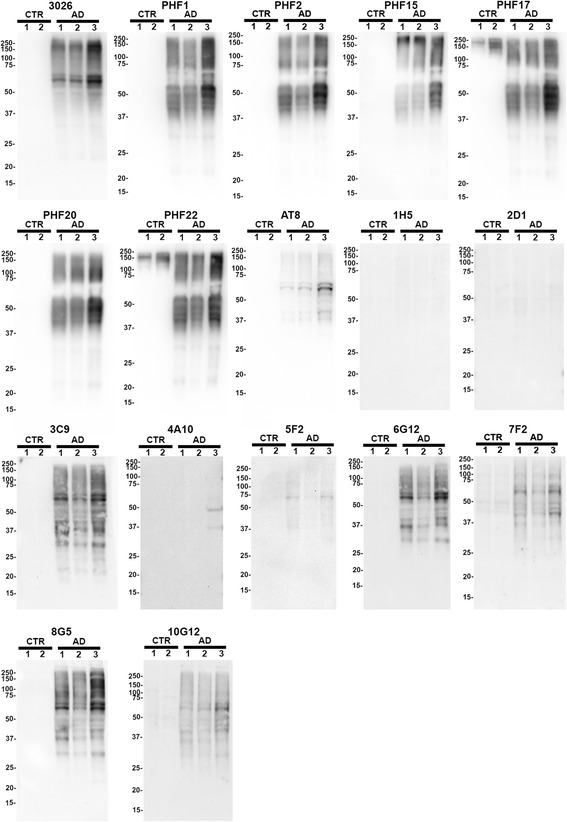
Characterization of the novel tau antibodies in detecting biochemically sarkosyl-insoluble tau in human brain lysates from AD patients. Immunoblotting analysis of the sarkosyl-insoluble fraction from the temporal cortex of human AD cases (*n* = 3) and control cases (CTR; *n* = 2). Samples were biochemically fractionated as described in “Material and Methods.” Equal amounts of proteins (10 μg) from each sample was resolved onto 10% polyacrylamide gels and analyzed by immunoblotting with each antibody indicated above *blot*, including total tau antibody 3026. The mobilities of molecular mass markers are shown on the *left*

The ability of the new monoclonal antibodies to detect sarkosyl-insoluble tau in the temporal cortex tissue from AD (*n* = 3) versus control (*n* = 2) cases was also assessed by immunoblotting (Fig. 6). Of the new PHF antibodies, PHF2, PHF15 and PHF20 specifically detect detergent insoluble tau only in the AD cases, while PHF17 and PHF22 showed some non-specificity in the control lanes, consistent with the immunoblot results from the mouse total brain lysates. The phospho-specific antibodies (3C9, 6G12, 7F2, 8G5, 10G12) generated to the AT8 epitope showed higher levels of detection of sarkosyl-insoluble tau in AD samples than our phospho-independent or phospho-selective antibodies (1H5, 2D1, 4A10, 5F2).

## Discussion

Previous studies have demonstrated that tau phosphorylation at the AT8 and PHF1 epitopes occur early in disease [8, 9, 37] and that these can be targets for immunotherapy [3, 4, 6, 7, 13, 22, 30, 45]. Here, we tried to generate new monoclonal antibodies similar to these epitopes and validated their specificities for tau and reactivity for pathological inclusions. In our epitope analysis studies, we included lysates from tau KO mice as a control to establish the level of specificity and cross-reactivity of the antibodies with other proteins in the brain homogenates. The apparent non-specificity of tau antibodies can typically arise from two main sources. First, because of the low amounts of phosphorylated tau present in a normal wild type mouse, anti-phospho-tau antibodies can tend to show increased non-specific cross-reactivity [41]. This can probably explain the significant cross-reactivity of the original AT8 clone [19] to multiple high molecular weight species shown here, present in both nTg and tau KO mice. None of the antibodies within our newly generated series, except for PHF 17 and PHF22, show detectable cross-reactivity with non-tau species. A second reported source of erroneous tau detection can arise from the presence of mouse Ig in the brain homogenates [41]. This apparent non-specificity of anti-tau antibodies in mouse homogenates can be due to the reactivity of the secondary anti-mouse IgG used for detection with endogenous Ig, which is approximately the same molecular mass as tau [41]. It is worthwhile to mention that none of the mice included in our study were perfused before harvesting brains. At least using our detection methods, we did not encounter any issues due to mouse Ig reactivity.

All of the newly characterized tau antibodies recognize both endogenous mouse tau as well as human 1 N/4R tau present in PS19 Tg mice. By immunoblotting analysis, the human tau expressed in PS19 mice [49] migrates slower (i.e. has an apparent larger molecular mass) than endogenous tau in nTg because these transgenic mice express the 1 N/4R human tau isoform, while 0 N/4R tau is the predominant isoform expressed in adult mouse brain [35].

Our screen for epitope specificity showed that PHF20 is specific for tau phosphorylated at S404, while our other new PHF antibodies (PHF2, PHF15, PHF17 and PHF 22; Table 1) are similar to PHF1, recognizing tau phosphorylation at both S396 and S404. All these antibodies show strong reactivity with tau in the sarkosyl-insoluble fractions of human AD temporal cortex, while PHF2, PHF15, and PHF20 show no cross-reactivity in the control samples. These antibodies will be used in future studies to compare the progressive pathological phosphorylation of one versus both of these phosphorylation sites.

**Table 1:** Summary of new tau monoclonal antibodies

Antibody	Antigen	Specificity	Isotype
PHF2	^391^EIVYKpSPVVSGDTpSPRHLS^409^	phosphorylated pS396/S404	IgG_1_
PHF15	^391^EIVYKpSPVVSGDTpSPRHLS^409^	phosphorylated pS396/S404	IgG_1_
PFH17	^391^EIVYKpSPVVSGDTpSPRHLS^409^	phosphorylated pS396/S404	nd
PHF20	^391^EIVYKpSPVVSGDTpSPRHLS^409^	phosphorylated S404	IgG_2B_
PHF22	^391^EIVYKpSPVVSGDTpSPRHLS^409^	phosphorylated S396/S404	IgG_1_
1H5	^193^DRSGYSpSPGpSPGpTPGSRSR^211^	prefers phosphorylated tau	IgM
2D1	^193^DRSGYSpSPGpSPGpTPGSRSR^211^	phosphorylation independent	IgG_1_
3C9	^193^DRSGYSpSPGpSPGpTPGSRSR^211^	prefers phosphorylated T205	IgG_1_
4A10	^193^DRSGYSpSPGpSPGpTPGSRSR^211^	phosphorylation-independent	IgG_1_
5F2	^193^DRSGYSpSPGpSPGpTPGSRSR^211^	phosphorylation-independent	IgG_1_
6G12	^193^DRSGYSpSPGpSPGpTPGSRSR^211^	phosphorylated T205	IgG_1_
7F2	^193^DRSGYSpSPGpSPGpTPGSRSR^211^	phosphorylated T205	IgG_1_
8G5	^193^DRSGYSpSPGpSPGpTPGSRSR^211^	phosphorylated T205	IgG_1_
10G12	^193^DRSGYSpSPGpSPGpTPGSRSR^211^	phosphorylated T205	IgG_1_

Listed are the antigens used to generate each antibody, the specificity for each antibody, and their isotypes

All of the antibodies generated in attempt to mimic the AT8 epitope were shown by immunoblotting to be relatively specific for tau and even more specific than the AT8 antibody. One set of antibodies are specific for phosphorylated T205, while another group are relatively phosphorylation independent (Table 1). In particular, one of these antibodies, clone 7F2, that was the best at revealing tau pathology in human tissue, is specific for tau phosphorylated at T205. While another antibody, clone 2D1, is phosphorylation-independent, reacting with both phosphorylated and non-phosphorylated tau. However, all of the new phospho-specific antibodies (3C9, 6G12, 7F2, 8G5, 10G12) generated against the AT8-like epitope showed robust detection of tau in the sarkosyl-insoluble samples of AD human brain tissue, while the phospho-independent antibodies (1H5, 2D1, 4A10, 5F2) displayed much weaker signal. In addition, the comparison of the sarkosyl-insoluble tau profiles detected by immunoblotting with the PHF antibodies relative to the phospho-specific antibodies raised against the AT8 epitope revealed marked differences. Immunoblotting patterns among the antibodies directed to the same epitope, however, were more conserved. These differences could be due to altered tau species with distinct phosphorylation and/or conformational properties or additional types of post-translational modifications such as a cross-linking and cleavage. Nevertheless, these data demonstrate the diverse nature of aggregated tau species even within the same brain samples.

There is mounting experimental evidence that tauopathies can progress by inter-cellular transmission prionoid mechanisms [23, 32, 36] and can be secreted in a diseased brain; consequently tau immunotherapies have been successful in mitigating or halting tauopathy in preclinical models [3–7, 12, 13, 22, 30, 44, 45, 48]. Indeed, one such humanized antibody (ABBV-8E12) has been approved to proceed to Phase 2 clinical trial in early AD and progressive supranuclear palsy patients (Clinical Trial # NCT02880956 and # NCT02985879). Given the enormous therapeutic promise for tau antibodies in patients and the fact that tauopathies are a wide spectrum of diseases, it is possible that we will need to tailor tau immunotherapy at different disease stages or in different tauopathy patients with antibodies that have avidity to progression-specific phosphorylation epitopes, disease-specific conformations, or even different antibody effector functions. At this time, it is unclear if phospho-independent or phospho-specific tau epitopes, and even which phosphorylation sites, might be more robust therapeutic targets. The new tau specific antibodies described here, some of which reveal diverse biochemical tau signatures, will allow further testing of these notions.

## Conclusions

We have generated and demonstrated the specificity of a series of new monoclonal antibodies recognizing tau phosphorylated at S396/S404, S404 or T205. Furthermore, we have established several new phosphorylation independent monoclonal antibodies against amino acid residues 193–211 in human tau. These antibodies will be useful in future studies of tau pathology progression and to experimentally devise immunotherapeutic intervention strategies.

## Abbreviations

AD: Alzheimer’s disease
BCA: Bicinchoninic acid
BSA: Bovine serum albumin
DAB: 3, 3′- diaminobenzidine
DMEM: Dulbecco’s Modified Eagle Medium
ELISA: Enzyme-linked immunosorbent assay
FBS: Fetal bovine serum
FTDP-17t: Frontotemporal dementia and parkinsonism linked to chromosome 17 with tau pathology
GSK3β: Glycogen synthase kinase 3β
HBSS: Hank’s balanced salt solution
HRP: Horseradish peroxidase
HS: High-salt
Ig: Immunoglobulin
IP: Intraperitoneal
KO: Knockout or null
MAPK: Mitogen-activated protein kinase
mcKLH: Mariculture keyhole limpet hemocyanin
NFTs: Neurofibrillary tangles
nTg: Non-transgenic
P301L: Proline 301 leucine
PBS: Phosphate buffered saline
S199A: Serine 199 alanine
S202A: Serine 202 alanine
S396A: Serine 396 alanine
S404A: Serine 404 alanine
SDS: Sodium dodecyl sulfate
T205A: Threonine 205 alanine
TBS: Tris-buffered saline
Tg: Transgenic
